# 

**Published:** 1913-08-16

**Authors:** 


### The Latest Treatment for Contracted Pelvis.

A most ingenious method of circumventing the dangers of delivery in women with contractions of the pelvis has lately been put into practice on the Continent, according to the *Medical Review*. A French obstetrician, Dr. Rotter, had a patient whose diagonal conjugate measured about three and three-quarter inches; the true conjugate was probably therefore not much over three inches. She had several pregnancies, but was unable to give birth to a living child. Rotter then decided to resect the promontory of the sacrum, and thus increase the conjugate diameter of the pelvis. He accordingly opened the abdomen and exposed the promontory by reflecting the peritoneum. The middle sacral artery was then ligatured; the projecting bone was shaved down with a large concave chisel, and trimmed with a smaller instrument. Speedy recovery followed, and it was found that the promontory had been replaced by a flat surface, and that the conjugate diameter had been increased by about three-fifths of an inch. The patient has not yet become pregnant again, but her future labour in such an event will be very interesting. It appears that Schmidt has already done this operation twice, in each case when doing a Cæsarean section necessitated by the contraction. In one case he increased the conjugate by two-fifths of an inch, and in another by slightly less; neither of the patients has yet been through a labour since the operation, so the practical test of this new idea is so far lacking.

### Appointments.

Mr. W. F. Haslam has been elected Joint Professor of Surgery at the University of Birmingham.

Mr. C. Stewart Wink has been appointed medical officer to the Halstead Union Workhouse.

Mr. Victor Vesselovsky has been appointed resident assistant anæsthetist to St. Mary's Hospital.

Dr. Payne, of Ardfin, Torquay, has been elected honorary surgeon to the Torbay Hospital, vice Dr. Arnold, resigned.

Dr. Hall Walshe has been appointed house surgeon at Torbay Hospital, Torquay, in place of Dr. J. Russell Orchard, resigned.

Dr. Frank Radcliffe has been appointed honorary acting surgeon to the Oldham Royal Infirmary, vice Dr. Robertson, retired.

Mr. J. R. Gyllencreutz has been appointed junior assistant medical officer at St. John's Hill Infirmary of the Wandsworth Union.

Dr. T. Shadick Higgins, assistant medical officer of health for Birmingham, has been appointed medical officer of health for St. Pancras in succession to the late Dr. Sykes.

Dr. A. J. Clark, assistant in the Department of Pharmacology at University College, has been appointed Lecturer in Pharmacology at Guy's Hospital Medical School. His successor at University College will be appointed at the beginning of next session.

Dr. G. H. Hunt, F.R.C.P., has been appointed assistant physician at Guy's Hospital, to fill the vacancy to be occasioned by the retirement of Dr. Newton Pitt, the senior member of the medical staff, and Mr. Harold Chapple, F.R.C.S., has been appointed obstetric surgeon, in place of the late Mr. J. H. Targett. Mr. W. H. Trethowan, F.R.C.S., has been elected surgeon in charge of the Orthopædic Department.

The retirement of Sir William Collins, M.D., F.R.C.S., from the active surgical staff of the London Temperance Hospital has caused Mr. H. J. Paterson, F.R.C.S., assistant surgeon, to be appointed full surgeon, whilst Mr. McAdam Eccles, F.R.C.S., of St. Bartholomew's Hospital, has been appointed a surgeon to the hospital. Mr. James McClure has been elected surgeon to the out-patients and Mr. J. S. Horsford, F.R.C.S., has been appointed ophthalmic surgeon to the hospital.

### DOCTORS' WILLS.

Dr. William Henry Eagland, M.D., of Scarborough, formerly of Harrogate, at one time house surgeon at the Hull General Infirmary, and for some years managing director of the West Yorkshire Iron and Coal Company, Limited, who died on April 17, aged eighty-one, left estate of the gross value of £34,009, of which £30,985 is net personalty.

### Personalia.

Mr. T. Wheeler has been elected a governor of Retford Hospital, Nottinghamshire, in place of Mr. J. A. H. Hirst, who has resigned.

Sir Rickman J. Godlee has been re-elected president and Mr. G. H. Makins, C.B., and Sir Frederic Eve were elected vice-presidents of the Royal College of Surgeons.

Queen Alexandra has become a life governor of the Royal Dental Hospital, Leicester Square, W.C., having allotted £200 from the Alexandra Day collection to that institution.

Mr. H. Work Dodd, F.R.C.S., has become consulting ophthalmic surgeon at the Royal Free Hospital, where Mr. J. G. French, F.R.C.S., has been appointed surgeon to the throat, nose, and ear department.

Queen Alexandra has expressed to the Lord Mayor, Lord Ninian Crichton-Stuart, M.P., Sir Marcus Samuel, and Sir Ernest Hatch, the administrative committee for distributing the money collected on Alexandra Day, her sincere thanks for their co-operation in making the awards.

The London County Council has appointed Dr. R. M. Beaton, Mr. Henry Mills, and Mr. Cuthbert Wilkinson to be additional members of the London Insurance Committee under Section 59 of the National Insurance Act. At the first meeting of the properly constituted Committee Mr. J. A. Dawes, M.P., was elected chairman and Mr. R. W. Moffrey vice-chairman.

### Gifts and Bequests.

Captain Thomas Jessop, of Huddersfield, has bequeathed £1,000 to the Huddersfield and Upper Agbrigg Infirmary.

Miss Eliza Anne Bateman, of Clifton, Bristol, bequeathed the residue of her estate, which will apparently amount to about £5,000, to Muller's Orphan Homes, Ashley Down, Bristol.

Queen Alexandra Nurses' Home.—Mr. Washington Singer has sent £100 for Queen Alexandra Nurses' Home at the Alton Cripples' Hospital, in response to the Archdeacon of London's appeal.

Mr. William Banks, of Dundee, has left to the Dundee Royal Infirmary, £1,050; Dundee Royal Victoria Hospital, £1,050; Dundee Convalescent Home, £1,000; and to the Dundee Sick Poor Nursing Society, £1,000.

The President of the Manchester Children's Hospital, Pendlebury, Sir George W. Agnew, Bart., M.P., is defraying the cost of the erection of a sea-wall around the convalescent branch at St. Anne's-on-the-Sea, Lancs., to protect the Home from the ravages of the sea.

Mrs. Almeric Paget, who has already given handsome contributions towards the new wards for children, Addenbrooke's Hospital, Cambridge, has just given £750 towards the new electric lift which it is proposed to have installed in the hospital, to serve not only the existing wards, but the new wards.

Florence Nightingale's Statue.—The Derby Corporation has voted £300 towards the cost of the stonework which will surround Countess Gleichen's statue of Florence Nightingale, to be erected as a county memorial in Derby. The board of the Derbyshire Royal Infirmary have provided the site, which will have a 40-feet frontage to London Road just inside their own grounds.

